# Study of Mesostructured CeO_2_ Synthesis via Nanocasting Using SBA-15 as a Template: Influence of the Cerium Precursor

**DOI:** 10.3390/ijms252313016

**Published:** 2024-12-03

**Authors:** Álvaro Moreno de la Calle, Arturo J. Vizcaíno, Alicia Carrero, José A. Calles, Pedro J. Megía

**Affiliations:** 1 Chemical and Environmental Engineering Group, Rey Juan Carlos University, Tulipan Street s/n., 28933 Mostoles, Madrid, Spain; alvaro.moreno@urjc.es (Á.M.d.l.C.); alicia.carrero@urjc.es (A.C.); joseantonio.calles@urjc.es (J.A.C.); pedro.megia@urjc.es (P.J.M.); 2 Institute of Sustainable Technologies, Rey Juan Carlos University, Tulipan Street s/n., 28933 Mostoles, Madrid, Spain

**Keywords:** ceria, structured materials, chemical synthesis, nanostructures

## Abstract

Mesoporous materials with high surface area, large pore volume, and adjustable pore size are promising in the fields of adsorption and heterogeneous catalysis. In this work, ordered mesoporous ceria structures were successfully prepared via nanocasting using SBA-15 as a template, with Ce(NO_3_)_3_·6H_2_O or CeCl_3_·7H_2_O as ceria precursors. The materials were characterized before and after template removal. The CeO_2_ crystallite size in the CeO_2_/SBA-15 composites increases with successive impregnations until it reaches the pore size of the SBA-15. Upon removal of the SBA-15 template, the synthesized materials exhibit pore diameters corresponding to the wall thickness of the SBA-15, evidencing that the inverted structure was obtained. Mesoporous ceria exhibits increasingly ordered structure up to five successive impregnations with 1.3 mmol_Ce_/g_SBA-15_. Using cerium chloride as a precursor, highly ordered structures were reached after only three impregnations. The feasibility of this synthesis in fewer steps (1, 3, and 5), employing the same amount of Ce precursor (6.7 mmol_Ce_/g_SBA-15_), was also studied. Results show a higher ordering degree and oxygen mobility capacity at higher impregnation steps. The mesostructured ceria samples exhibit significantly higher oxygen mobility than commercial bulk ceria, along with high thermal stability, which highlights the usefulness of these structures.

## 1. Introduction

Mesoporous materials are considered one of the most promising materials in heterogeneous catalysis due to their high surface area, large pore volume, and narrow pore size distribution between 2–50 nm [1]. They can be used in many catalytic applications, such as contaminants removal from gas–liquid industrial effluents by adsorption or for the selective separation of different compounds [2]. In this regard, mesostructured materials with ordered pore distribution have some advantages compared to mesoporous materials such as better control over pore size and pore distribution. This would result in enhanced physical and chemical properties such as thermal stability, mechanical strength, and adsorption capacity.

By using cationic surfactants—with positively charged hydrophilic “head” groups and a hydrophobic “tail” that forms micelles—as templates in mesoporous material synthesis, the size of their pores can be easily controlled by employing compounds with molecular sizes smaller than the pore size, allowing them to access and diffuse into their structure [3]. Mesoporous silica structures have been widely reported for different purposes. For example, SBA-16 is used in the catalytic conversion of epoxides to cyclic carbonates by reacting with CO_2_ due to its thicker wall framework, small particle size, and three-dimensional cage structure, which allows higher thermal stability and large pore volume [4]. MCM-41 is used as an adsorbent for various types of compounds, whether organic, inorganic, or gases [5]. SBA-15 has cylindrical mesopores interconnected by micropores and has been used as a catalyst support, improving active phase dispersion and reactant molecule diffusion [6,7].

On the other hand, despite their non-porous structure, metal oxides find numerous applications in the field of catalysis. For example, Fe_2_O_3_ is used in the Fischer–Tropsch reaction and in the ethyl benzene dehydrogenation to styrene; TiO_2_ is widely used in photocatalysis for wastewater treatment and air purification through NO_x_ removal, and Cu–Cr oxides are commonly used as catalysts for hydrogenation or combustion reactions [8]. Concretely, CeO_2_ has multiple applications in heterogeneous catalysis due to its redox properties and high oxygen mobility, such as photocatalysis [9], reforming processes [10], biomedical applications [11], and energy storage [12,13], among others. However, its application in catalysis can be constrained by its limited surface area due to a non-porous structure [14]. For that reason, the synthesis of ordered mesoporous metal oxides has attracted considerable attention from both the scientific and technological communities. This heightened interest is the result of their capability to interact with molecules, ions, and atoms, not only on the external surface but also within the internal porous structure [15,16]. In this regard, there are different methods to produce porous metal oxides, such as soft-templating, hard-templating, and colloid crystal templating methods [17]. Soft-templating methods are best suited for synthesizing mesoporous metal oxides with an amorphous or semi-crystalline framework [18]. The hard-templating method, also known as nanocasting, allows the preparation of crystalline porous metal oxides by using structured mesoporous materials as templates [19]. On the contrary, the colloidal crystal templating method can be used in the synthesis of three-dimensional ordered structures by self-assembling colloidal particles, which are typically employed to achieve highly ordered macroporous structures [20]. These methods can allow the synthesis of ceria nanoparticles with different mesoporous structures and morphologies. However, there is a deficiency in achieving high-quality materials concerning thermal stability, pore size distribution, and crystallinity, especially for mesostructured material [21].

Based on the foregoing, this work proposes a study of the synthesis of mesostructured CeO_2_ prepared by nanocasting using SBA-15 as a hard template. The effect of the Ce precursor (nitrate or chloride) and the number of synthesis steps on the achievement of a well-ordered porous structure with high oxygen mobility capacity have been studied. 

## 2. Results and Discussion

### 2.1. Effect of the Ceria Precursor

#### 2.1.1. Characterization of the CeO_2_/SBA-15 Composites

**Figure 1:**
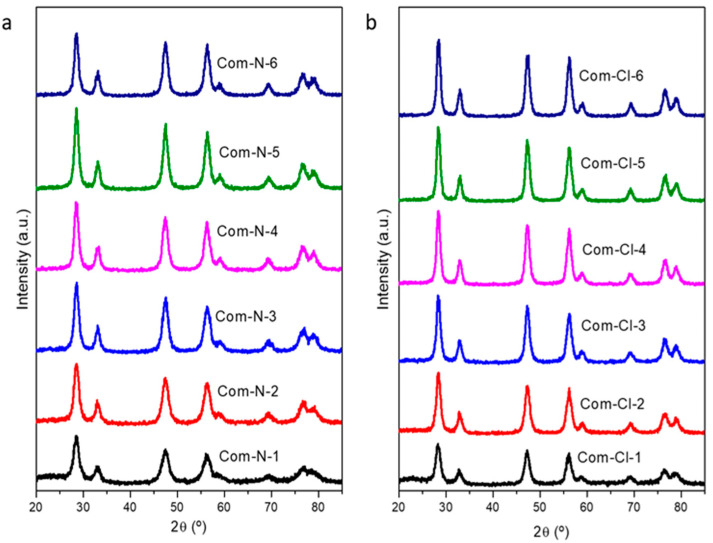
XRD patterns of the calcined composites using Ce(NO_3_)_3_·6H_2_O (**a**) and CeCl_3_·7H_2_O (**b**) as ceria precursors.

The presence of cerium oxide in the calcined CeO_2_/SBA-15 composites was verified by XRD analysis. In this regard, Figure 1 displays the diffractograms of the calcined CeO_2_/SBA-15 composites. In all the samples, the characteristic peaks of cubic CeO_2_ (JCPDS 01-089-8436) arise at 2θ = 28.5, 33.1, 47.5, 56.3, 59.1, 69.5, 76.6, and 79.1°, ascribed to the (111), (200), (220), (311), (222) (400), (331), and (420) planes, respectively. Mean crystallite sizes were calculated by applying the Debye–Scherrer equation to the (111) plane for all the samples, which corresponds to the main diffraction line of the CeO_2_ pattern. The estimated crystallite sizes are summarized in Table 1. From these results, it is possible to appreciate that the mean CeO_2_ crystallite size grows with the number of impregnations as the amount of Ce incorporated increases. This value increases up to almost 10 nm, which indicates that the CeO_2_ was filling the SBA-15 pores, given that the maximum in the BJH pore size distribution of the commercial SBA-15 used as the template was 10.4 nm. Similar crystallite sizes were obtained with both precursors, although slightly higher values were observed when cerium chloride was used for SBA-15 impregnation. This difference could be explained by the kinetics of the precursor’s thermal decomposition. The bond between cerium and chlorine in cerium chloride is more stable than the cerium–nitrogen bond in cerium nitrate. Consequently, a higher activation energy is required to initiate bond cleavage in cerium chloride, resulting in decelerated thermal degradation kinetics. This was corroborated by the TGA analysis of both precursors (Figure S1) and would explain the larger crystal sizes of CeO_2_ crystallites in composites prepared from cerium chloride.

**Figure 2:**
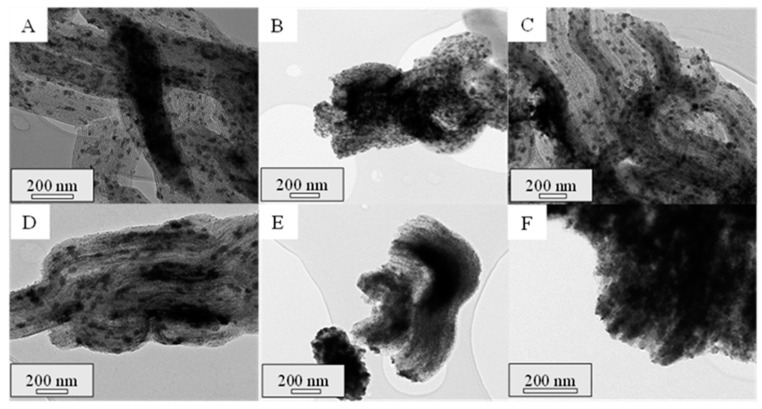
TEM micrographs of the calcined composites Com-N-1 (**A**), Com-N-2 (**B**), Com-N-3 (**C**), Com-N-4 (**D**), Com-N-5 (**E**), and Com-N-6 (**F**).

**Figure 3:**
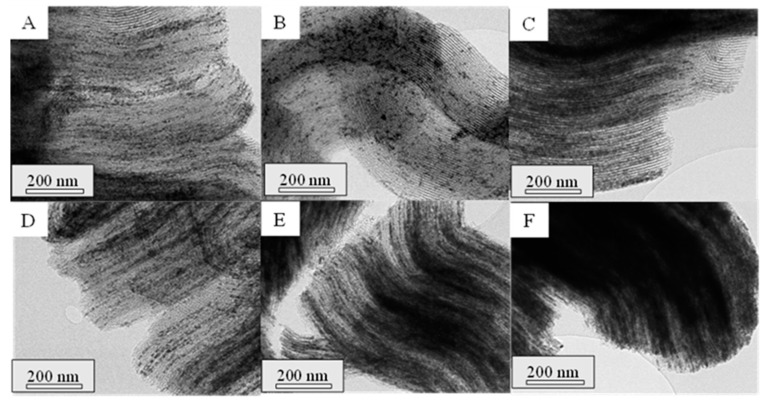
TEM micrographs of the calcined composites Com-Cl-1 (**A**), Com-Cl-2 (**B**), Com-Cl-3 (**C**), Com-Cl-4 (**D**), Com-Cl-5 (**E**), and Com-Cl-6 (**F**).

All the calcined composites were further characterized by TEM. In this regard, the micrographs of the successive impregnations can be seen for the samples prepared with Ce(NO_3_)_3_·6H_2_O and CeCl_3_·7H_2_O in Figure 2 and Figure 3, respectively. All the composites showed the highly ordered hexagonal mesoporous structure typical of SBA-15, with supported cerium oxide particles (dark zones) with different shapes: irregular extended particles that seem to adapt to the porous structure or round-shape particles that may be in the external surface of the support. It is noteworthy that, in both cases, the amount of cerium oxide increases with each successive impregnation, filling the pores of the SBA-15 material. As a result, there is a progressive growth of the oxide crystallites, corroborating the mean crystallite sizes discussed above. Moreover, differences in the CeO_2_ particle distribution within the SBA-15 channels can be distinguished. When using CeCl_3_·7H_2_O, cerium oxide particles seem to be more elongated and better distributed compared to the use of Ce(NO_3_)_3_·6H_2_O. In this regard, Wang et al. [22] reported different shapes in ceria nanocrystals prepared through a precipitation-hydrothermal method without surfactant or template when using cerium chloride and cerium nitrate as precursors. They observed that the presence of Cl^−^ ions led to the formation of CeO_2_ nanowires, given their higher tendency to form complexes with cerium ions or surface complexes with ceria compared to NO_3_^−^, while ceria nanoparticles with round shapes were obtained when using cerium nitrate, in line with the results obtained in the present work.

**Figure 4:**
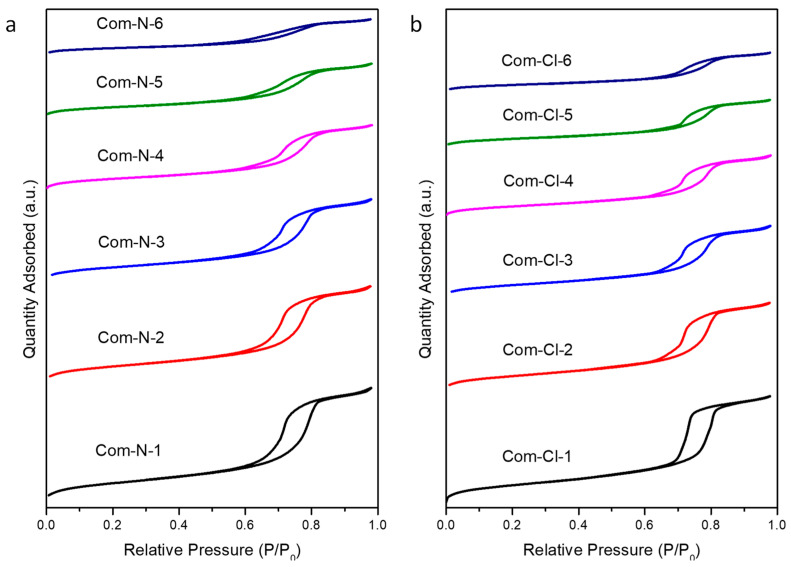
Nitrogen adsorption/desorption isotherms of the calcined composites using Ce(NO_3_)_3_·6H_2_O (**a**) and CeCl_3_·7H_2_O (**b**) as ceria precursors.

**Figure 5:**
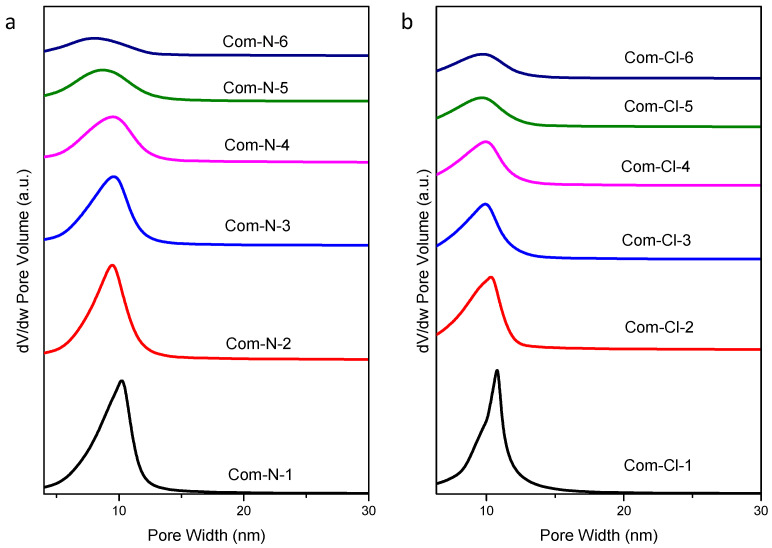
Pore size distribution of the calcined composites using Ce(NO_3_)_3_·6H_2_O (**a**) and CeCl_3_·7H_2_O (**b**) as ceria precursors.

The Nitrogen adsorption/desorption isotherms of the different composites with successive impregnations using Ce(NO_3_)_3_·6H_2_O and CeCl_3_·7H_2_O as ceria precursors are represented in Figure 4. As can be discerned, a type IV isotherm with an H1 hysteresis loop, according to the IUPAC classification, can be observed in all the samples. This is evidence of the well-ordered hexagonal mesoporous structure of SBA-15 used as the template [23]. It is noteworthy that the shape of the isotherms obtained from both precursors changes progressively with successive impregnations, given that ceria is effectively filling the SBA-15 pores, thus decreasing its BET surface area (see Table 1). These facts demonstrate changes in the porosity, although the overall well-ordered mesoporous structure typical of SBA-15 is retained. Furthermore, as the number of impregnations increases, a broader pore size distribution is observed, as shown in Figure 5.

#### 2.1.2. Characterization of Replicated Mesoporous CeO_2_

The prepared composites were introduced into a NaOH bath, as described in the experimental section, to remove the SBA-15 used as a template. Then, the mesoporous ceria particles were recovered by filtration, washed with deionized water, and calcined at 600 °C. This procedure was possible only for the prepared structures after the 3rd impregnation since, for the 1st and the 2nd impregnations, the obtained particles were too small to be retained in the filter (pore size 0.45 μm). This could be due to incomplete filling of the micropores in the SBA-15, hindering connection between some of the nanowires, or to the low filling of the pores causing the CeO_2_ crystals to lack contact with each other.

**Figure 6:**
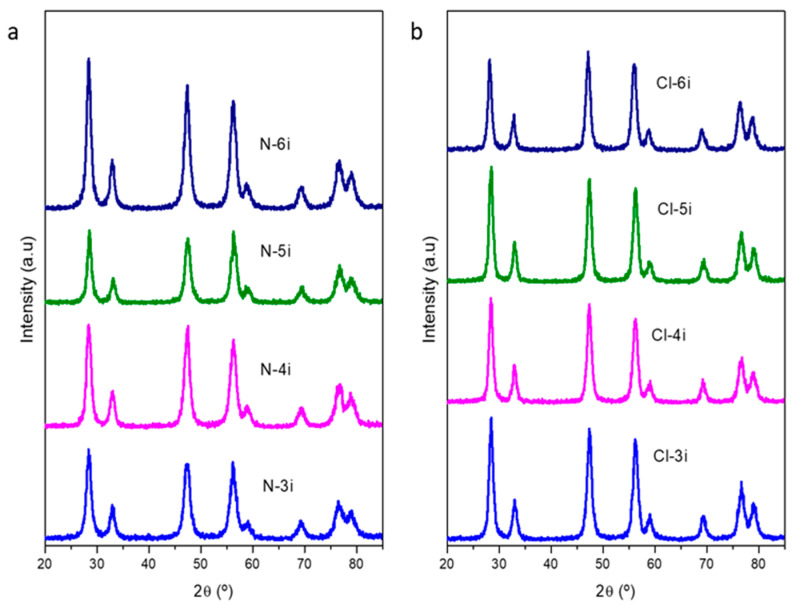
XRD patterns of the synthesized mesoporous ceria after successive impregnations using Ce(NO_3_)_3_·6H_2_O (**a**) and CeCl_3_·7H_2_O (**b**) as ceria precursors.

Figure 6 shows the XRD patterns of the synthesized mesoporous ceria obtained with 3 to 6 impregnations. Peaks show reflections at 2θ = 28.5, 33.1, 47.5, 56.3, 59.1, 69.5, 76.6, and 79.1° due to the presence of CeO_2_ with a cubic structure (JCPDS 01-089-8436) as in the case of the composites. By applying the Debye–Scherrer equation to the full-width-half-maximum diffraction line (2θ = 28.5°), mean crystallite sizes were calculated, and the obtained results are summarized in Table 2. It is noteworthy that the mean crystallite sizes increase with successive impregnations using cerium nitrate and cerium chloride as precursors, up to values around 10,4 nm, matching with the pore diameter of the original SBA-15 used as the template, as expected for a successful synthesis by nanocasting [24]. Knowing that cerium oxide is in its cubic structure and that the wavelength of the X-rays is λ = 0.15418 nm, the unit cell size was calculated using Bragg’s Law equation to analyze the crystalline structure of the prepared materials compared to the CeO_2_ pattern value. The results show that, for all the prepared materials from both nitrate and chloride, the calculated unit cell size was around 0.542 nm, close to the expected value for the CeO_2_ pattern (a = 0.541 nm), indicating that the crystal structure of the prepared materials was perfectly preserved.

**Figure 7:**
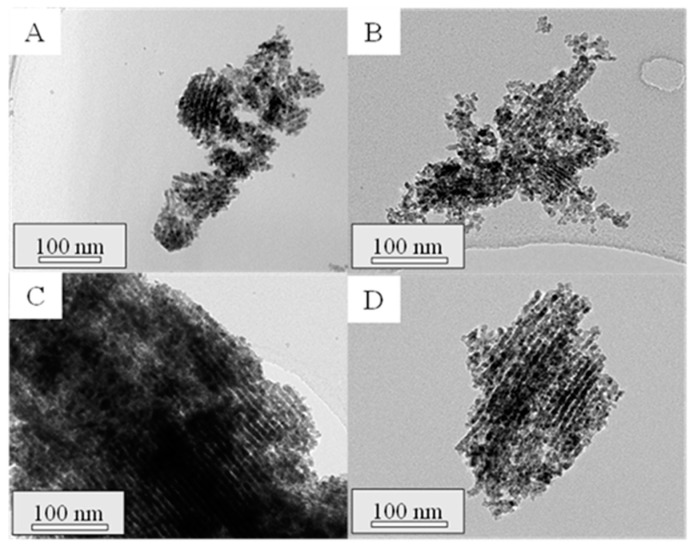
TEM micrographs of the synthesized mesoporous ceria, N-3i (**A**), N-4i (**B**), N-5i (**C**), N-6i (**D**).

**Figure 8:**
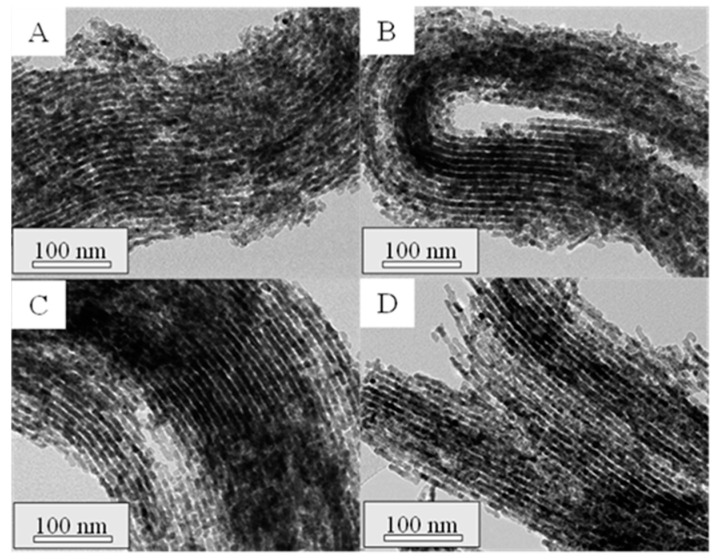
TEM micrographs of the synthesized mesoporous ceria Cl-3i (**A**), Cl-4i (**B**), Cl-5i (**C**), Cl-6i (**D**).

To assess the structure of the synthesized materials, TEM analyses were performed on the mesoporous ceria after the successive three to six impregnations. TEM micrographs are shown in Figure 7 and Figure 8 for the prepared materials using Ce(NO_3_)_3_·6H_2_O and CeCl_3_·7H_2_O as ceria precursors, respectively. In the micrographs acquired for the mesoporous ceria synthesized from cerium nitrate, smaller particles with a lower ordering degree can be appreciated. This fact can explain the slightly higher BET surface areas of these samples (Table 2) given the available interparticle space between the smaller mesoporous ceria particles. On the contrary, it is possible to observe highly ordered structures when using cerium chloride even at the 3rd impregnation (Cl-3i sample), achieving shapes similar to those expected for SBA-15. This is in line with the results achieved for the composites in which the use of cerium chloride as a ceria precursor seems to result in a better distribution of CeO_2_ particles within the SBA-15 pores (see Figure 3). Some defective ceria nanorods can be distinguished around the shape of the mesostructured ceria particles in the Cl-3i sample, which progressively disappear as the number of impregnations increases up to the 5th impregnation (Cl-5i sample) where an ordered structure without defects is achieved.

**Figure 9:**
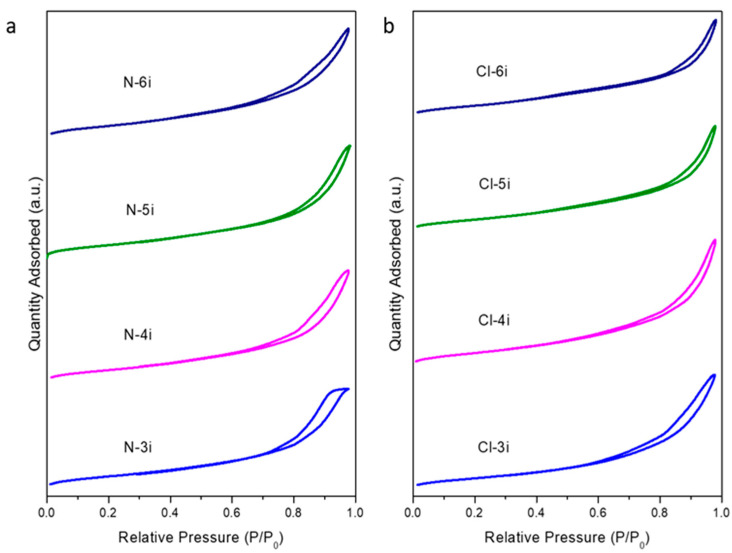
Nitrogen adsorption/desorption isotherms of the synthesized mesoporous ceria after successive impregnations using Ce(NO_3_)_3_·6H_2_O (**a**) and CeCl_3_·7H_2_O (**b**) as ceria precursors.

**Figure 10:**
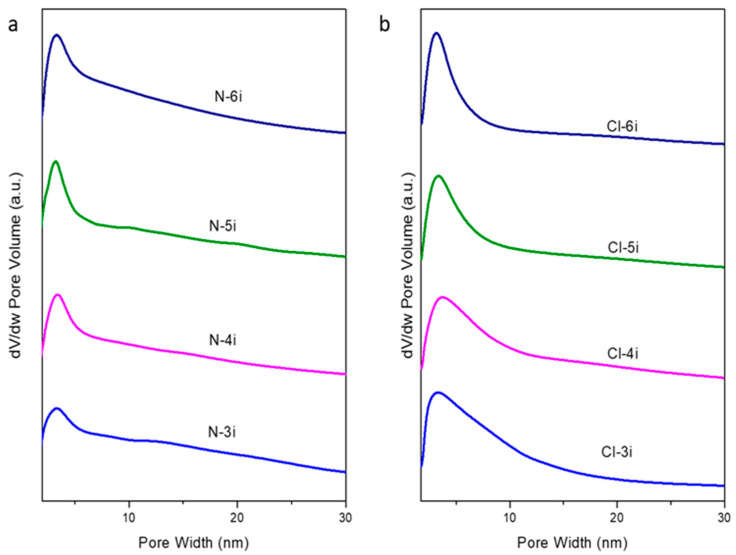
Pore size distribution of the synthesized mesoporous ceria after successive impregnations using Ce(NO_3_)_3_·6H_2_O (**a**) and CeCl_3_·7H_2_O (**b**) as ceria precursors.

Thus, N_2_-physisorption isotherms of the mesoporous ceria after 3, 4, 5, and 6 impregnations are shown in Figure 9. All the samples showed type IV isotherms with H3-type hysteresis loops according to the IUPAC classification [25] in contrast to the H1-type hysteresis loop achieved with the CeO_2_/SBA-15 composites, which is characteristic of the SBA-15 material used as the template. This indicates that there is no adsorption limitation in the last interval of P/P_0_, which is typical of layered particles that give rise to porosity as described elsewhere [14]. Table 2 summarizes the textural properties of the synthesized samples. Similar BET surface areas were obtained with both ceria precursors, with slightly higher values for the samples prepared using Ce(NO_3_)_3_·6H_2_O. On the other hand, pore diameters from the maximum BJH distribution (Figure 10) are close to 3.5 nm independently of the ceria precursor, which agrees with the wall thickness of the SBA-15 used as the template, typically around 4 nm [24]. Concerning the pore size distribution, it is possible to observe that the pore distribution of the N-6i sample exhibits a broader distribution than the Cl-6i sample. This suggests different porosities in the sample, which could be ascribed to smaller mesoporous ceria particles being formed when using ceria nitrate as the precursor. In fact, estimations by dynamic light scattering demonstrate that, in all cases, for the same number of impregnations, the average particle size is smaller when using cerium nitrate instead of cerium chloride (see Table 2).

### 2.2. Effect of the Number of Steps During the Synthesis

**Figure 11:**
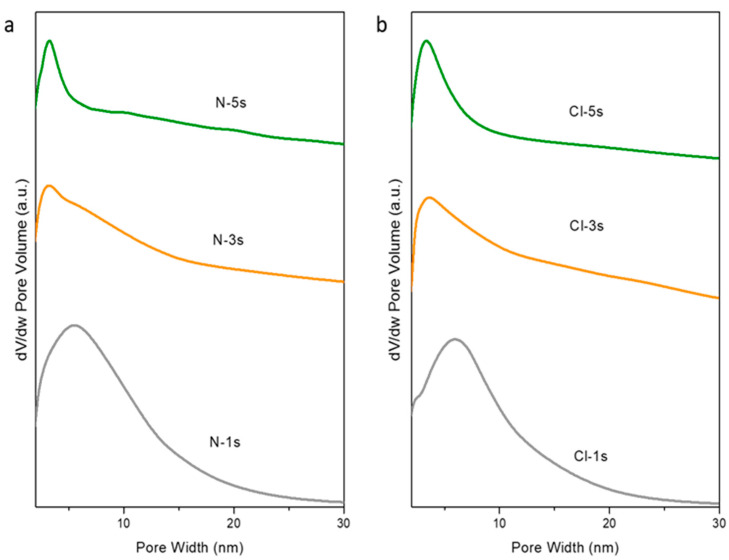
Pore size distribution of the mesoporous ceria synthesized in one, three, and five steps using (**a**) Ce(NO_3_)_3_·6H_2_O and (**b**) CeCl_3_·7H_2_O as precursors.

**Figure 12:**
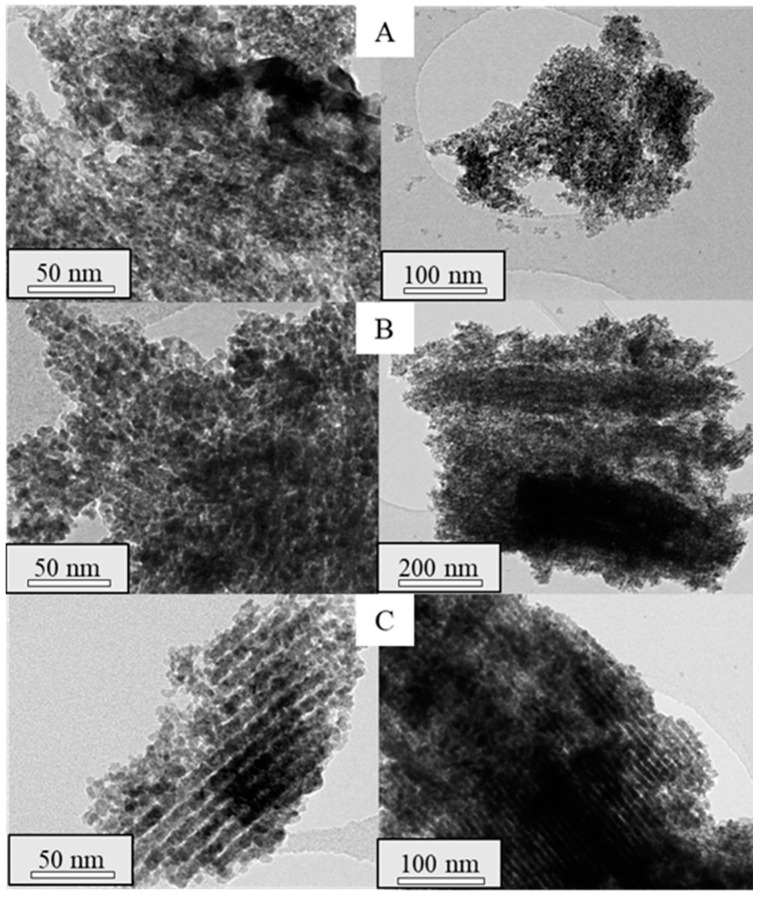
TEM micrographs of the synthesized mesoporous ceria N-1s (**A**), N-3s (**B**), N-5s (**C**).

**Figure 13:**
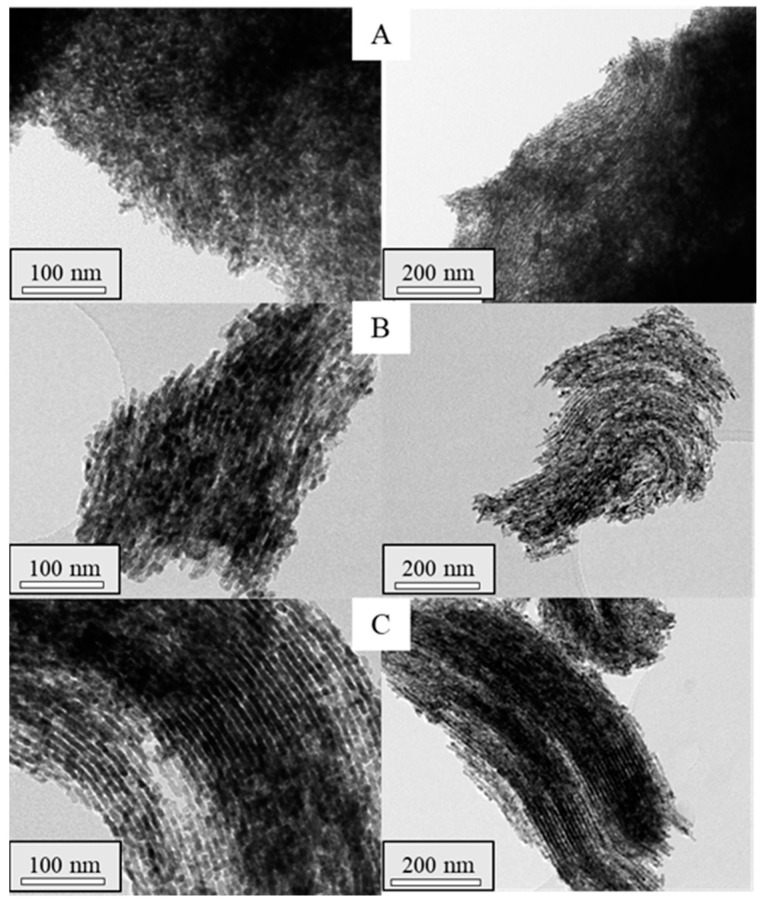
TEM micrographs of the synthesized mesoporous ceria Cl-1s (**A**), Cl-3s (**B**), Cl-5s (**C**).

Based on the above, five impregnations were enough to obtain highly ordered mesostructured ceria. To simplify the synthesis procedure, new materials were synthesized using the total cerium content of the previous procedure with five impregnations (6.7 mmolCe/gSBA-15), but carrying out the synthesis in one and three steps. The textural properties and the particle size of these materials are summarized in Table 3 along with the mean crystallite size estimated by applying the Debye–Scherrer equation to the main diffraction line of the CeO_2_ pattern. The obtained results revealed that, for both precursors, the synthesis of mesoporous ceria in one step resulted in the highest BET surface areas and particle size, while the lowest crystallite sizes were obtained. This fact could be due to a higher concentration of cerium salts during the synthesis procedure which may hinder the diffusion of cerium into the SBA-15 pores, leading to larger particles with less-ordered structures. In this regard, Figure 11 shows that, as the number of steps in the synthesis increases, a narrower pore size distribution is achieved, which implies a better arrangement of the structure. This was also corroborated by TEM images (Figure 12 and Figure 13 for samples prepared with Ce(NO_3_)_3_·6H_2_O and CeCl_3_·7H_2_O as cerium precursors, respectively). Concerning the average particle size, the materials prepared from chloride again reached higher values in all the cases for the same number of synthesis steps. It can be noticed that particle size decreases with the number of steps during the synthesis because the addition of the cerium precursor in fewer steps results in a higher concentration in the solution, facilitating the formation of agglomerates hindering the formation of a well-ordered structure.

**Figure 14:**
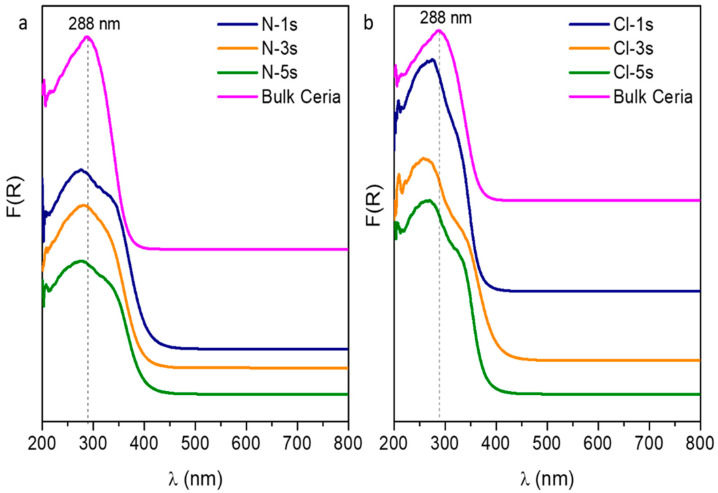
UV-vis spectra of the Bulk Ceria and the mesoporous CeO_2_ using (**a**) Ce(NO_3_)_3_·6H_2_O and (**b**) CeCl_3_·7H_2_O as ceria precursors.

Furthermore, the oxygen mobility of the materials synthesized in one, three, and five steps was evaluated through ultraviolet-visible spectrophotometry (UV-vis) and temperature-programmed desorption of CO_2_ (CO_2_-TPD). In this regard, Figure 14 shows the UV-vis spectra for each sample. The spectrum of commercial bulk ceria is included for comparative purposes. Whereas the spectrum of bulk ceria (non-porous ceria) presents a peak at λ = 288.3 nm, the prepared mesoporous materials show a shift towards shorter wavelengths (“blue-shift”), indicating a higher presence of Ce^3+^, which may result in higher oxygen vacancies (19). Concretely, the spectra of samples prepared with cerium chloride as the precursor, especially the Cl-5s sample, show significant displacement corresponding to the presence of Ce^3+^ that produces defects on the ceria surface, thus increasing the oxygen vacancies [26]. These results agree with those obtained from CO_2_-TPD (Figure S2), given that the total consumption for N-5s and Cl-5s samples was 77.0 and 81.4 μmol_CO2_/g, respectively, while the total consumption for bulk ceria was 9.5 μmol_CO2_/g. According to Liu et al. [27], CO_2_ adsorption ability in CO_2_-TPD perfectly aligns with the variations in the concentration of surface oxygen vacancies, so the higher CO_2_ consumption in Cl-5s is indicative of higher oxygen mobility. Broadly, the increased oxygen mobility over the mesostructured ceria compared to bulk ceria makes its application interesting for multiple fields in heterogeneous catalysis since higher oxygen mobility would favor redox reactions.

**Figure 15:**
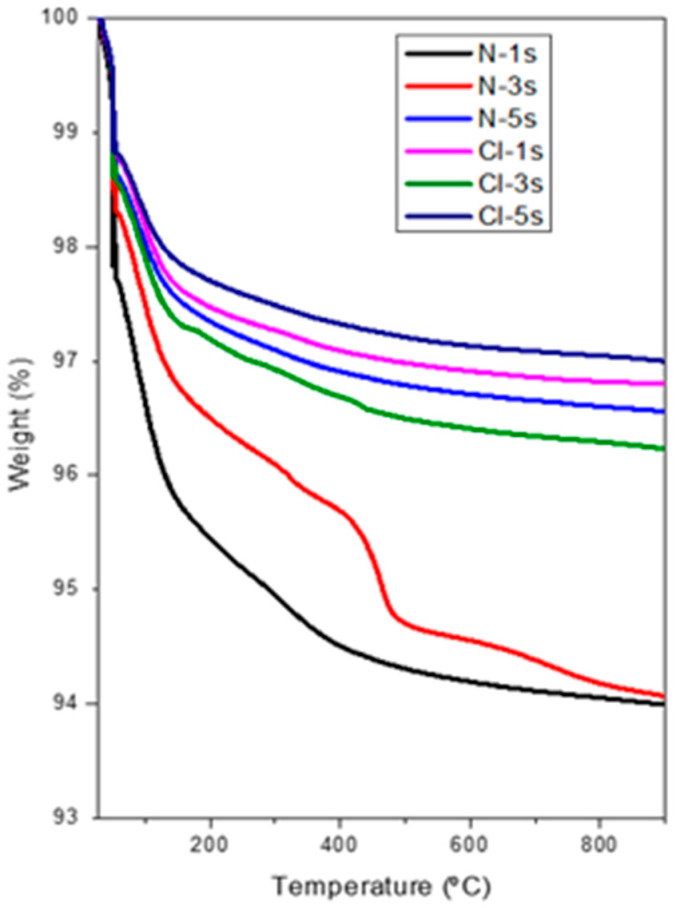
Thermogravimetric analysis of N-1s, N-3s, N-5s, Cl-1s, Cl-3s, Cl-5s.

Finally, the thermal stability of mesoporous CeO_2_ was analyzed by thermogravimetric analysis in air up to 900 °C with a heating rate of 5 °C/min. The results are displayed in Figure 15 as the weight loss % versus temperature. These curves show weight loss lower than 6 wt.% up to 900 °C in all cases, confirming the high thermal stability of the prepared materials, although the weight loss was even lower in samples synthesized using cerium chloride as a ceria precursor.

**Figure 16:**
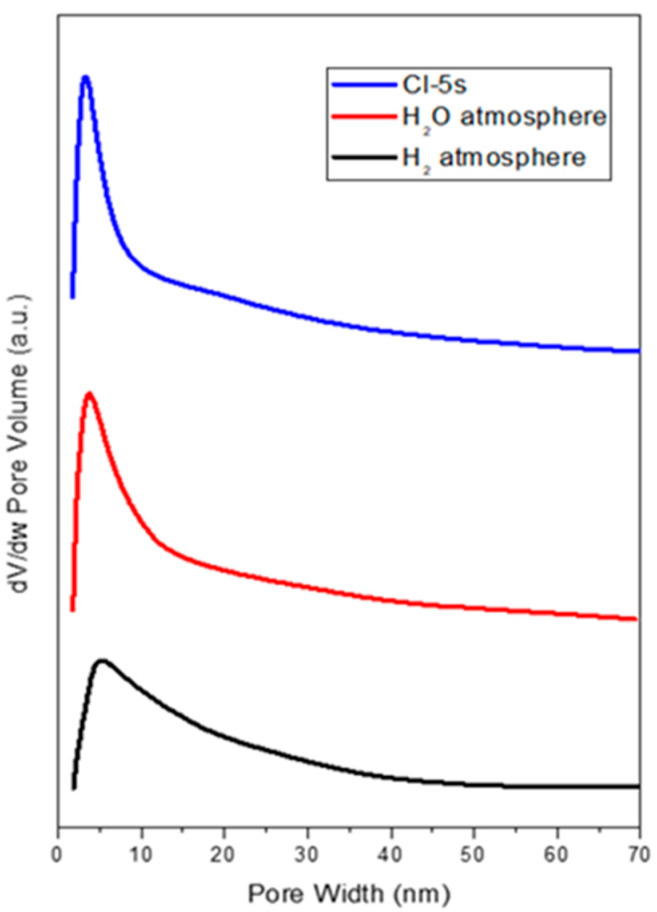
Pore size distribution of Cl-5s under different atmospheres at 600 °C: hydrogen and water.

Based on the above, the Cl-5s sample has reached a highly-ordered ceria mesostructure with high oxygen mobility, making it promising for multiple redox applications. Thus, finally, the stability of this material was tested at 600 °C under different atmospheres (hydrogen and water). To evaluate the effect of these thermal treatments on the structure of Cl-5s, N_2_-physisorption analyses were subsequently performed. In this regard, Figure 16 shows the pore size distribution according to the BJH method. It is noteworthy how the pore size distribution changed under the different atmospheres tested. While under the water (steam) atmosphere, the distribution scarcely changed compared to the original Cl-5s, indicating the preservation of the ordered structure. However, under reducing conditions with hydrogen, the distribution broadened significantly, indicating some distortion in the structure. This effect may be related to the increasing oxygen vacancies generated when Ce^4+^ is reduced to Ce^3+^. In this regard, Lee et al. [28] reported the effect of ceria reduction at different temperatures. During the reduction, they observed a lower proportion of Ce^4+^, which was being reduced to Ce^3+^, leading to nonstoichiometric ceria (CeO_2−x_). This nonstoichiometric ceria contains oxygen vacancies on the ceria surface that would change the crystal structure. This effect was more pronounced at temperatures above 600 °C, in which the Ce^3+^ proportion increased from 20.8% (data for bulk ceria) to 29.1% at 700 °C. These results would explain the change in the pore size distribution when Cl-5s is thermal-treated under H_2_-atmosphere. Despite this, Cl-5s material kept its ordered structure differently, thus highlighting that synthesized mesostructured ceria can have a wide range of possibilities in heterogeneous catalysis.

## 3. Materials and Methods

The mesoporous CeO_2_ samples were prepared by incorporating Ce(NO_3_)_3_·6H_2_O (Scharlau, Barcelona, Spain) or CeCl_3_·7H_2_O (Thermo Scientific, Waltham, MA, USA) as ceria precursors into the pores of a commercial SBA-15 (ACS Material) as a template. A typical synthesis undergoes as follows: the proper amount of the corresponding precursor to achieving a CeO_2_/SBA-15 ratio of 1.3 mmolCe/gSBA-15 was dissolved in 50 mL of pure ethanol (Scharlau). Then, 6 g of SBA-15 was gradually added to the solution. After obtaining a homogeneous mixture, the ethanol was completely evaporated by combining stirring at 60 °C with ultrasonication. Subsequently, it was calcined under static air at 600 °C for 6 h using a heating ramp of 2 °C/min to convert all the cerium precursor embedded in the SBA-15 channels into the metal oxide, CeO_2_. This procedure was repeated up to six times to increase ceria loading in the prepared composites. Finally, to remove the SBA-15 used as the template, the calcined composites were dissolved in a 2 M solution of NaOH (Panreac, Castellar del Vallès, Spain) and kept under stirring for 4 h at 60 °C. Then, mesoporous cerium particles were recovered by filtration, washed with distilled water until a neutral pH was achieved, and subsequently calcined in an air stream at 600 °C. Upon determining the optimum number of impregnations, the procedure was repeated using the total amount of cerium precursor to achieve ordered structures through a reduced number of steps, thereby simplifying the synthesis process.

Samples were named Com-X-Y for the composites, where X corresponds to the precursor (Cl for cerium chloride and N for cerium nitrate), and Y refers to the number of impregnations (from 1 to 5). Similarly, after removing the SBA-15 template, samples were denoted as X-Yz where z corresponds to “i” for materials prepared after successive impregnations, or “s” for samples prepared in fewer steps (one, three, and five) employing the same amount of cerium precursor used in the 5-steps synthesis.

All the prepared samples (both CeO_2_/SBA-15 composites and the final ceria materials) were characterized using different techniques.

XRD diffractograms were acquired on a Philips X’pert PRO diffractometer using Cu Kα radiation to ensure the presence of cerium oxide and calculate its crystallite size by applying the Scherrer equation to the main diffraction line of the CeO_2_ pattern.

The pore size distribution, specific surface area, and pore volume were determined from N_2_ adsorption–desorption isotherms at 77K on a Micromeritcs Tristar 3000 analyzer. Specific surface areas were calculated using the Brunauer–Emmett–Teller method (BET), and pore volume was estimated from the desorption isotherm by applying the Barret–Joyner–Halenda method (BJH).

To obtain information about the morphology of the prepared materials, TEM analysis was performed. Micrographs were obtained on a JEOL JEM 2100 (200 kV) microscope with a resolution of 0.25 nm at the National Centre for Electron Microscopy (CNME, Complutense University of Madrid). Before the analysis, samples were suspended in butanol by ultrasonication and subsequently deposited on a carbon-coated copper grid.

The thermal stability of the synthesized samples was analyzed through thermogravimetric analysis in airflow up to 1000 °C with a heating ramp of 5 °C/min on a TA Instrument thermobalance SDT 650.

A Particle Size Analyzer NanoPlus-3 by dynamic light scattering (DLS) was used to evaluate the particle size distribution. For that, samples were previously dispersed in the ethanol as a solvent (1 mg CeO_2_/mL ethanol).

The oxygen mobility capacity of the ceria materials was determined by a Cary Series UV-vis-NIR spectrophotometer in a wavelength range of 800 to 200 nm and an Autochem 2930 to perform a temperature-programmed desorption of CO_2_ (TPD).

## 4. Conclusions

The successful optimization of the synthesis of ordered mesoporous ceria structures was achieved in this study through nanocasting. This process utilized SBA-15 as the template and involved successive impregnations of Ce(NO_3_)_3_·6H_2_O or CeCl_3_·7H_2_O as ceria precursors using a 1.3 mmolCe/gSBA-15 ratio in each step. Based on the characterization results, it was revealed that the size of the CeO_2_ crystallites within the prepared composites increased proportionally to the number of impregnations, eventually reaching diameters similar to the pore size of the SBA-15 used as the template. Upon removal of the template, the pore diameter of the synthesized materials corresponded with the wall thickness of the initial material, indicating the achievement of the inverted structure of SBA-15. Interestingly, these materials exhibited increasingly ordered structures with each successive impregnation. This effect was more pronounced in the 5-step synthesis. Furthermore, the use of cerium chloride as the precursor enabled a better particle distribution throughout the templated structure, resulting in highly ordered structures even for 3-step synthesis. In contrast, the use of cerium nitrate led to mesoporous ceria with a higher available specific surface area due to the smaller particle size achieved. This emphasizes the importance of precursor selection in tailoring the properties of mesoporous ceria materials; for example, using cerium nitrate, smaller particles were obtained compared to the use of chloride because it decomposes more easily. Besides, the mesostructured ceria synthesized by nanocasting showed enhanced oxygen mobility compared to bulk ceria and high thermal stability under different atmospheres. These results demonstrate that the prepared materials are suitable for a wide variety of applications in heterogeneous catalysis and adsorption to eliminate contaminants from industrial liquid or gas effluents, owing to their ordered structure and high surface area.

## Figures and Tables

**Table 1:** Physical and textural properties of the calcined composites using Ce(NO_3_)_3_·6H_2_O and CeCl_3_·7H_2_O as ceria precursors.

Sample	S_BET_ (m^2^/g)	D_pore_ (nm) ^a^	D CeO_2_ (nm) ^b^
Com-N-1	369	10.4	6.6
Com-N-2	326	9.6	6.9
Com-N-3	281	9.9	8.0
Com-N-4	240	9.4	8.6
Com-N-5	200	8.0	9.2
Com-N-6	144	7.9	9.3
Com-Cl-1	398	10.4	6.4
Com-Cl-2	337	10.3	7.0
Com-Cl-3	281	10.1	8.0
Com-Cl-4	243	10.4	8.8
Com-Cl-5	197	9.8	9.4
Com-Cl-6	161	9.7	9.5

^a^ Calculated through the maximum of the BJH pore size distribution. ^b^ Calculated from the reflection peak (111) of the CeO_2_ pattern in the calcined composites using the Debye-Scherrer equation.

**Table 2:** Physical and textural properties of the synthesized mesoporous ceria after successive impregnations using Ce(NO_3_)_3_·6H_2_O and CeCl_3_·7H_2_O as precursors.

Sample	S_BET_ (m^2^/g)	D_pore_ (nm) ^a^	D CeO_2_ (nm) ^b^	Particle size (nm) ^c^
N-3i	121	3.4	9.1	159.0
N-4i	120	3.5	9.5	158.3
N-5i	121	3.2	10.2	196.4
N-6i	123	3.2	10.4	233.1
Cl-3i	112	3.5	9.3	214.8
Cl-4i	116	3.5	9.9	221.2
Cl-5i	106	3.5	10.3	233.4
Cl-6i	106	3.5	10.5	243.6

^a^ Calculated through the maximum of the BJH pore size distribution. ^b^ Calculated from the reflection peak (111) of the CeO_2_ pattern in the calcined samples using the Debye-Scherrer equation. ^c^ Average particle size determined from dynamic light scattering using ethanol as the solvent.

**Table 3:** Physical and textural properties of the synthesized mesoporous ceria in one, three, and five steps using Ce (NO_3_)_3_·6H_2_O and CeCl_3_·7H_2_O as precursors.

Sample	S_BET_ (m^2^/g)	D CeO_2_ (nm) ^a^	Particle Size (nm) ^b^
N-1s	188	7.3	544.4
N-3s	144	7.9	255.3
N-5s	121	10.4	196.4
Cl-1s	147	7.6	602.4
Cl-3s	110	8.2	260.7
Cl-5s	106	10.5	233.4

^a^ Calculated from the reflection peak (111) of the CeO_2_ pattern using the Debye–Scherrer equation. ^b^ Average particle size determined from dynamic light scattering using ethanol as the solvent.

## Data Availability

Dataset available on request from the authors.

## Supplementary Materials

The following supporting information can be downloaded at: https://www.mdpi.com/article/10.3390/ijms252313016/s1.
