# Supplementary material for: Study of Mesostructured CeO2 Synthesis via Nanocasting Using SBA-15 as a Template: Influence of the Cerium Precursor
Source: Int J Mol Sci. 2024 Dec 3;25(23):13016. doi: 10.3390/ijms252313016 (PMC11641326; doi:10.3390/ijms252313016)
Supplement: Supplementary file 1 [file ijms-25-13016-s001.zip › ijms-3339237-supplementary.pdf]

International Journal of
Molecular Sciences

MDPI

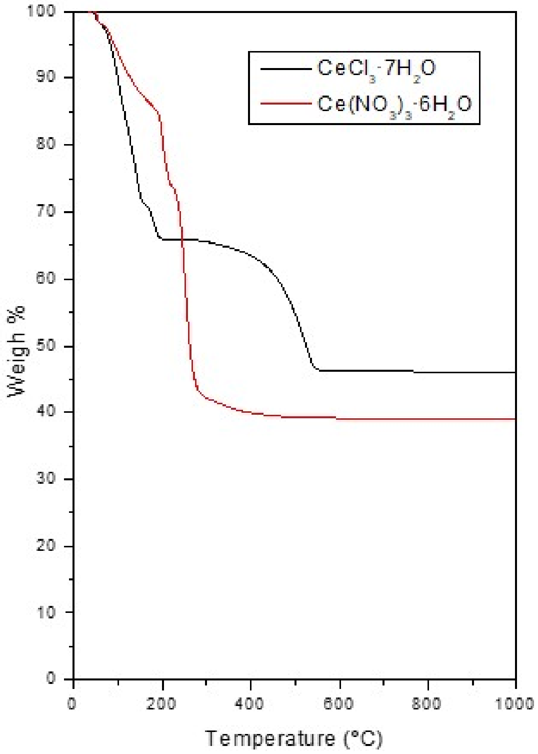

Figure S1. Thermogravimetric analyses of ceria precursors: $CeCl_3·7H_2O$ and $Ce(NO_3)_3·6H_2O$

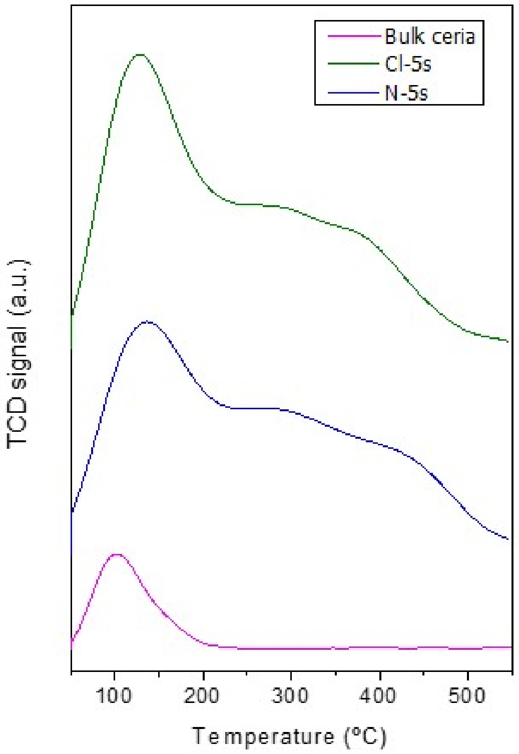

Figure S2. CO_2-TPD of bulk ceria and mesoporous CeO_2 using $Ce(NO_3)_3·6H_2O$ and $CeCl_3·7H_2O$.
